# Oxidative Stress‐Associated Alterations in Sperm ACTL7A Expression and Function in Men With Varicocele

**DOI:** 10.1155/omcl/1776251

**Published:** 2026-08-13

**Authors:** Sahar Tahamtan, Marziyeh Tavalaee, Mohsen Forouzanfar, Mojtaba Jafarinia, Mohammad H. Nasr-Esfahani

**Affiliations:** ^1^ Department of Animal Biotechnology, Reproductive Biomedicine Research Center, Royan Institute for Biotechnology, ACECR, Isfahan, Iran, acecr.ac.ir; ^2^ Department of Biology, Marvdasht Branch, Islamic Azad University, Marvdasht, Iran, azad.ac.ir; ^3^ Pooyesh & Rooyesh Fertility Center, Isfahan Health Center, Isfahan, Iran

**Keywords:** ACTL7A, chromatin packaging, oxidative stress, sperm DNA fragmentation, varicocele

## Abstract

**Background:**

Actin‐like 7A (*ACTL7A*) is a testis‐specific cytoskeletal protein essential for spermiogenesis, acrosome formation, and oocyte activation. Given its critical role in sperm integrity and fertilization, ACTL7A protein may be particularly susceptible to oxidative and thermal stress associated with varicocele. This study aimed to evaluate *ACTL7A* gene, and protein expression in the sperm of infertile men with varicocele.

**Methods:**

Semen samples from infertile men with varicocele (*n* = 35) and fertile controls (*n* = 35) were assessed for standard sperm parameters (WHO 2021), redox status (2′,7′‐dichlorodihydrofluorescein diacetate [DCFH‐DA] and BODIPY probes), chromatin packaging (Chromomycin A3 [CMA3] and Aniline Blue staining), DNA integrity (Sperm Chromatin Structure Assay [SCSA]), *ACTL7A* gene, and protein expression (quantitative real‐time PCR and Western blotting).

**Results:**

Compared to fertile controls, the varicocele group exhibited significantly lower sperm quality, elevated oxidative stress, increased lipid peroxidation, and greater chromatin immaturity and DNA fragmentation (*p* < 0.01). Notably, *ACTL7A* gene and ACTL7A protein expression levels were significantly reduced (*p* < 0.05), with inverse correlations observed between *ACTL7A* gene and markers of chromatin packaging defects, including protamine deficiency and residual histones (*p* < 0.05).

**Conclusion:**

*ACTL7A* gene expression and ACTL7A protein are significantly reduced in sperm from men with varicocele, likely causing defective chromatin remodeling and abnormal sperm head formation. This study first links varicocele‐associated oxidative stress to *ACTL7A*, suggesting its potential as a biomarker of testicular dysfunction. Future research should explore whether interventions like antioxidant therapy, varicocelectomy, or varicocele severity and duration improve sperm quality and reproductive outcomes.

**Summary**

• Actin‐like 7A (ACTL7A) expression is significantly reduced at both mRNA and protein levels in sperm from infertile men with varicocele.
• Elevated oxidative stress markers—including reactive oxygen species (ROS) and lipid peroxidation—are correlated with increased DNA fragmentation, residual histones, and protamine deficiency.
• Reduced ACTL7A expression is closely linked to defective chromatin packaging, impaired nuclear maturation, and abnormal sperm morphology.

## 1. Introduction

The perinuclear theca (PT) is a detergent‐ and salt‐resistant structure surrounding the sperm nucleus, located between the inner acrosomal membrane and the nuclear envelope in the subacrosomal layer (SAL) and between the plasma membrane and nuclear envelope in the postacrosomal region (PAR). SAL proteins mainly originate from acrosomal vesicles, whereas PAR proteins are transported via the manchette. Unlike classical cytoskeletal structures, the PT contains unique spermatid‐ and sperm‐specific proteins and contributes to nuclear remodeling, acrosome–nucleus adhesion, chromatin stabilization, and potentially oocyte activation and early embryonic signaling [1, 2]. The inner acrosomal membrane–acroplaxome–nuclear envelope complex is formed through coordinated interactions among these compartments. SPACA1 on the inner acrosomal membrane likely binds the acroplaxome protein Actin‐like 7A (ACTL7A) to anchor the acrosome to the nucleus, and loss of either disrupts acrosomal attachment during spermatogenesis [3–5]. Calicin further supports this structural network, and its deficiency results in sperm DNA damage and fertilization failure, underscoring its essential role in sperm integrity [6].

ACTL7A encodes an actin‐like protein that is essential for spermatogenesis. The ACTL7A protein is primarily expressed in the testis. It plays a pivotal role in the later stages of sperm development, particularly in spermiogenesis, during which round spermatids transform into elongated spermatozoa. ACTL7A is implicated in cytoskeletal organization, which is necessary for sperm tail formation and motility. Additionally, it contributes to acrosome biogenesis, a crucial process for fertilization, as the acrosome contains enzymes required for oocyte penetration. Emerging evidence suggests that ACTL7A may serve as a biomarker for predicting fertilization outcomes in assisted reproductive technology (ART), with lower ACTL7A levels correlating with reduced embryo quality and poorer ART success rates [7].

Mutations or variations in the *ACTL7A* gene can lead to impaired spermatogenesis, culminating in subfertility or infertility in males. Animal model studies, particularly those involving mice with *Actl7A* mutations, have revealed defects in acrosome formation and postacrosomal structures, compromising sperm fertilization potential and embryonic development [3, 5, 8, 9]. These findings highlight the indispensable role of ACTL7A in normal testicular function and male fertility.

The perinuclear matrix hosts essential fertilization proteins, including PLCζ encoded by the *PLCZ1* gene in the postacrosomal region. Altered PLCζ expression or mislocalization impairs embryonic development and fertility. *ACTL7A* mutations increase postacrosomal thickness and reduce PLCζ, particularly in the equatorial region critical for oocyte fusion, leading to fertilization failure in humans and mice [3, 5, 8, 9].

Oxidative and thermal stress are interconnected factors that profoundly affect gene and protein expression, especially in sperm, which have minimal cytoplasm, limited antioxidant defenses, and high polyunsaturated fatty acid content, making them prone to lipid peroxidation and DNA damage. Spermatogenesis is highly temperature‐sensitive; even mild testicular hyperthermia, as in varicocele, disrupts gene/protein expression, cytoskeletal integrity, and mitochondrial function [10]. Heat stress destabilizes proteins, causing misfolding and aggregation, while oxidative stress further modifies amino acids, impairing proteostasis and protein function [11]. Mitochondrial dysfunction induced by heat increases reactive oxygen species (ROS) production, damaging proteins, lipids, and DNA [12]. In varicocele, impaired venous drainage leads to chronic hyperthermia and ROS accumulation, creating a hostile environment for germ cells and disrupting spermatogenesis, cytoskeletal organization, and sperm chromatin integrity, which may explain the high DNA fragmentation observed in infertile men [13–16].

Oxidative and thermal stress profoundly affect gene and protein expression during spermatogenesis. Considering the oxidative and thermal stress observed in varicocele, it is important to assess whether these conditions impair *ACTL7A* gene expression and ACTL7A protein function. Our previous work showed that PLCζ, encoded by *PLCZ1*, is significantly reduced in men with varicocele at both transcriptional and translational levels [17]. Building on this, the present study compares ACTL7A expression in sperm from infertile men with varicocele and fertile controls.

## 2. Materials and Methods

### 2.1. Ethical Considerations and Consent

This study was approved by the Royan Institute’s Ethics Review Board under the ethical code IR.ACECR.ROYAN.REC.1402.021. Prior to participation, all individuals provided written informed consent.

### 2.2. Study Design

The study included 35 semen samples from infertile men diagnosed with grade II or III varicocele based on Dubin and Amelar’s classification [18]. An additional 35 samples were obtained from fertile men without varicocele, whose semen parameters fell within the normal range and who had fathered at least one child. Semen samples were collected from individuals attending the Pooyesh Fertility Center. Participants were required to maintain an abstinence period of 2–7 days before providing semen samples at the andrology laboratory for analysis. Half of each sample was used to assess sperm parameters and function according to the WHO‐2021 guidelines [19], while the remaining portion was allocated for molecular analyses, including RNA and protein extraction for Western blot and quantitative real‐time PCR (qPCR). ACTL7A protein expression was analyzed by Western blot in sperm samples from four fertile men and 10 infertile men with varicocele, whereas gene expression was evaluated at the RNA level in all samples (*N* = 35) using qPCR.

### 2.3. Inclusion and Exclusion Criteria

#### 2.3.1. Inclusion Criteria

##### 2.3.1.1. Varicocele Group

Infertile men aged 20–45 years who were clinically diagnosed with grade II or III varicocele according to Dubin and Amelar’s classification were included in the study. At least one semen analysis was required for enrollment.

##### 2.3.1.2. Fertile Control Group

Fertile men aged 20–45 years with normal semen parameters based on WHO (2021) reference values and no clinical varicocele were included. Proven fertility within the past 12 months (pregnancy of partner) was required.

#### 2.3.2. Exclusion Criteria

Exclusion criteria were applied equally to both groups unless otherwise specified. Participants were excluded if they had any additional condition that could affect fertility—other than varicocele in the varicocele group—including leukocytospermia, azoospermia, severe oligozoospermia (<1 million sperm per ejaculate), seminal antisperm antibodies, genital or urogenital infections, anatomical abnormalities (except varicocele in the varicocele group), endocrine disorders such as hypogonadism or Klinefelter syndrome, a history of scrotal trauma or surgery, fever within the preceding 90 days, cancer or systemic disease, previous testicular sperm extraction or epididymal sperm aspiration, use of cryopreserved semen, or age >45 years. Additionally, in the fertile control group, men with clinical varicocele or abnormal semen parameters were excluded. The mean ± standard deviation (SD) of age and BMI for each study group was presented in Table 1, demonstrating the homogeneity of participants. In addition, information regarding potential confounding factors—including obesity (BMI ≥ 30 kg/m^2^), current smoking, alcohol or recreational drug use, and occupational exposure to potentially harmful agents—was collected through a questionnaire. Participants with these factors were excluded. Occupational exposures considered included continuous heat (e.g., bakers, furnace workers, and heavy vehicle drivers) and toxic substances (e.g., pesticides, solvents, and industrial chemicals).

**Table 1:** Baseline demographic and clinical characteristics of the study population.

Parameter	Varicocele group	Control group	*p*‐Value
Age (years) (mean ± SD)	34.17 ± 6.35 *N* = 35	33.11 ± 6.5 *N* = 35	0.5
BMI (kg/m^2^), (mean ± SD)	25.7 ± 2.8 *N* = 25	24.9 ± 3.1 *N* = 30	0.62
Smoking	Excluded		—
Alcohol consumption	Excluded		—
Occupational exposure	Excluded		—

### 2.4. Semen Analysis

Sperm concentration was determined by performing duplicate counts using a Neubauer sperm counting chamber (Sperm Meter, Sperm Processor; Garkheda, India). A 10 µL aliquot of liquefied semen was placed into the chamber for assessment. If sperm concentration was high, a 1:10 dilution was prepared using a 1% formalin solution in sodium bicarbonate. The final concentration was recorded in millions per microliter. Sperm motility was evaluated using a computer‐assisted sperm analysis (CASA) system (VideoTesTSperm 2.1; Russia). Morphological assessment was carried out through Diff‐Quik staining, and at least 100 spermatozoa were examined at high magnification (×1000) under bright‐field microscopy. The results were expressed as percentage of abnormal sperm morphology.

### 2.5. ROS Evaluation

ROS levels in spermatozoa were evaluated using the cell‐permeable dye 2′,7′‐dichlorodihydrofluorescein diacetate (DCFH‐DA) (Sigma–Aldrich, MO, USA). A 0.5 μM concentration of DCFH‐DA was introduced into the sperm suspension (1 × 10^6^ sperm/mL in PBS) and incubated at 37°C for 30 min. Fluorescence was measured via a FACSCalibur flow cytometer, and data were analyzed using CellQuest Pro and WinMDI 2.9 software. Results were expressed as the percentage of fluorescent spermatozoa [20].

### 2.6. Sperm Lipid Peroxidation Evaluation

Sperm lipid peroxidation was quantified using the BODIPY 581/591 C11 assay. Briefly, spermatozoa (2 million per ml cells) were incubated with BODIPY 581/591 C11 (D3861, Molecular Probes) at a final concentration of 5 μM for 30 min at 37°C. The samples were then washed twice with PBS before analysis. The proportion of spermatozoa exhibiting lipid peroxidation (BODIPY‐positive cells) was determined using a FACSCalibur flow cytometer (Becton Dickinson, San Jose, CA, USA). Results were expressed as the percentage of sperm lipid peroxidation.

#### 2.6.1. Sperm Protamine Deficiency Evaluation

To assess protamine deficiency, two fixed sperm smears were prepared for each sample using Carnoy’s solution. Each smear was treated with 200 μL of chromomycin A3 (CMA3) staining solution (0.25 mg/mL), followed by three washes with PBS. At least 200 sperm cells were analyzed under an epifluorescence microscope (Olympus, Japan) equipped with 460–470 nm filters at × 100 magnification. Spermatozoa deficient in protamine appeared light yellow, while those with normal protamine levels exhibited dark yellow fluorescence, as described by Simon et al. [21]. Results were expressed as the percentage of sperm protamine deficiency.

### 2.7. Sperm Histone Content Evaluation

For histone content evaluation, two sperm smears per sample were fixed with 3% glutaraldehyde and subsequently stained with 5% aqueous aniline blue dye in 4% acetic acid. The slides were then sequentially dehydrated in ethanol solutions (70%, 96%, and 100%) before being immersed in xylene for 5 minutes. Coverslips were mounted using Entellan. A minimum of 200 spermatozoa were randomly examined under an optical microscope, with blue‐stained spermatozoa indicating nuclear immaturity [21]. Results were expressed as the percentage of sperm residual histone in sperm.

### 2.8. Sperm Chromatin Structure Assay (SCSA)

The SCSA was conducted based on established guidelines by Evenson [22]. Following sperm concentration determination, 2 × 10^6^ sperm cells were suspended in TNE buffer (50 mM Tris‐HCl, pH 7.4; 100 mM NaCl; 0.1 mM EDTA; Merck, Darmstadt, Germany) to a final volume of 1 mL. A 1/5 portion of this suspension underwent SCSA, where 400 μL of acid‐detergent solution was added, followed by 1.2 mL of acridine orange (AO) staining solution (Sigma, St. Louis, USA) for 30 s. Samples were analyzed using a FACSCalibur flow cytometer (BD Biosciences, San Jose, CA, USA), with at least 10,000 sperm cells evaluated. The DNA Fragmentation Index (DFI %) was used to report the results.

### 2.9. RNA Extraction and cDNA Synthesis

Total RNA was extracted from sperm pellets using YTzol Pure RNA (YT9064, Yekta Tajhiz Azma, Iran) following the manufacturer’s guidelines. The integrity of the isolated RNA was verified via agarose gel electrophoresis, and its concentration was measured using a NanoDrop 1000 spectrophotometer (Thermo Scientific, USA) at 260 nm. To eliminate any residual DNA contamination, RNA samples were treated with RNase‐free DNase I (Thermo Scientific, USA). First‐strand cDNA synthesis was carried out using the PrimeScript II 1st Strand cDNA Synthesis Kit (TaKaRa, Japan).

### 2.10. qPCR

For qPCR analysis, cDNA templates were combined with SYBR Premix Ex Taq II (TaKaRa, Japan) in a final reaction volume of 10 μL. Reactions were performed in triplicate using specific primers. The thermal cycling conditions included an initial denaturation at 95°C for 30 s, followed by 40 cycles of denaturation at 95°C for 5 s, annealing at 56°C for *ACTL7A* and 60°C for GAPDH for 10 s, and extension at 72°C for 30 s. The reactions were conducted using a StepOnePlus Real‐Time PCR System (Applied Biosystems, USA). Gene‐specific primers for *ACTL7A* and *GAPDH* were designed using Beacon Designer 8.13 (Premier Biosoft, USA) and validated for secondary structures and specificity using Oligo 7.56 software (Molecular Biology Insights, USA). ACTL7A gene expression levels were normalized to GAPDH, and relative quantification was performed using the 2^−ΔΔCt^ method. Each sample was analyzed in three technical replicates. Fold change values for the experimental group were expressed relative to the mean of the fertile control group, which was set to 1. This ensures that the control group serves as a baseline for comparison of gene expression levels between experimental and control samples [23, 24].

#### 2.10.1. Primer Sequences

***GAPDH***: Forward: 5′‐ AACAGCCTCAAGATCATCAGC ‐3′; Reverse: 5′‐ ATGAGTCCTTCCACGATACCAA ‐3′ and,

***ACTL7A*:** Forward: 5′AGTGAGCATACGATCCGCTAC‐3′; Reverse: 5′‐ATGGCTTGAAGAACATCTCCGA‐3′.

### 2.11. Sperm ACTL7A Protein Expression

ACTL7A protein expression was examined in sperm samples from infertile men with varicocele (*N* = 10) and fertile men (*N* = 4) using the Western blot technique at SARA Laboratory (Tabriz, Iran). Protein was extracted from sperm pellets using TRIzol reagent (Sigma–Aldrich, USA), and protein concentration was quantified using Bradford’s assay (Bio‐Rad, USA). Samples (12 μL per lane) were separated on 10% sodium dodecyl sulfate‐polyacrylamide gels (SDS‐PAGE) (SinaClon, Iran) and transferred onto polyvinylidene difluoride (PVDF) membranes (Bio‐Rad, USA). To prevent nonspecific binding, membranes were blocked with 2% skimmed milk (Merck, USA) for 75 min. Membranes were incubated with primary antibodies against ACTL7A (Proteintech, Catalog No: 17355–1‐AP) and GAPDH (Santa Cruz Biotechnology, Catalog No: sc‐32233), followed by washes and incubation with secondary antibodies [(mouse anti‐rabbit IgG‐HRP, Santa Cruz Biotechnology, Catalog No: sc‐2357) and (m‐IgGκBP‐HRP, Santa Cruz Biotechnology, Catalog No: sc‐516102)]. After additional washes, protein bands were visualized using an enhanced chemiluminescence (ECL) system (Santa Cruz, USA) as per the manufacturer’s instructions. Protein expression was normalized to GAPDH, and densitometric analysis was performed using ImageJ software. For Western blot analysis, fertile samples (*n* = 4) were run in duplicate, while each varicocele sample (*n* = 9) was analyzed once due to limitations in sample availability, antibody cost, and time, and all blots were performed following standard procedures.

### 2.12. Statistical Analysis

All statistical analyses were conducted using SPSS software (version 22). Normality of the data was assessed using the Shapiro–Wilk test. Comparisons between groups were made using an independent *t*‐test, with statistical significance set at *p* < 0.05. Results are reported as mean ± SD. The correlation between study parameters was assessed using Pearson’s correlation coefficient.

## 3. Results

**Figure 1:**
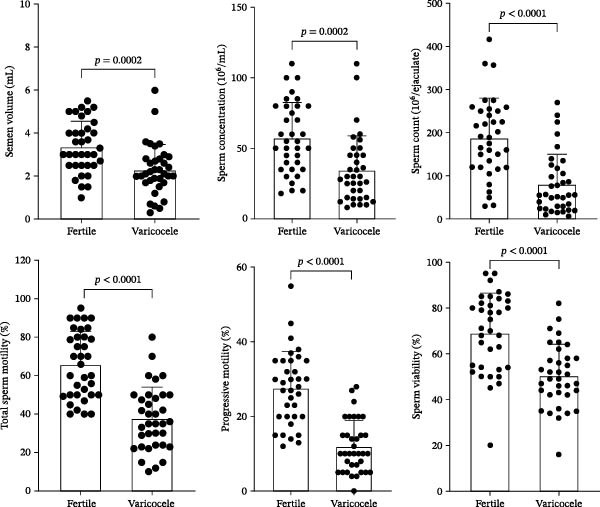
Comparison of sperm parameters between infertile men with varicocele and fertile controls. Data are presented as mean ± standard deviation (SD), with individual data points shown as scatter plots. Comparisons between groups were performed using an independent *t*‐test. *p*‐Values < 0.05 were considered statistically significant.

**Figure 2:**
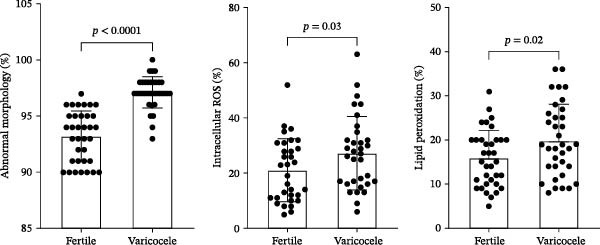
Comparison of sperm abnormal morphology and oxidative stress between infertile men with varicocele and fertile controls. Data are presented as mean ± standard deviation (SD), with individual data points shown as scatter plots. Comparisons between groups were performed using an independent *t*‐test. *p*‐Values < 0.05 were considered statistically significant.

**Figure 3:**
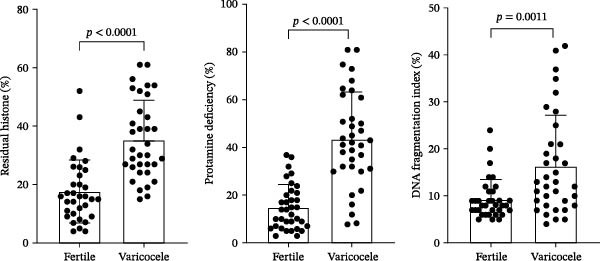
Comparison of sperm chromatin status between infertile men with varicocele and fertile controls. Data are presented as mean ± standard deviation (SD), with individual data points shown as scatter plots. Comparisons between groups were performed using an independent *t*‐test. *p*‐Values < 0.05 were considered statistically significant.

In this study, semen samples were analyzed from two groups: 35 infertile men diagnosed with varicocele and 35 fertile men without varicocele. The average age of men in both groups was comparable, with no statistically significant difference between them (34.96 ± 5.6 versus 33.5 ± 6.18; *p* = 0.31). As illustrated in Figure 1, several sperm parameters—including semen volume, sperm concentration, total sperm count, motility, progressive motility, and sperm viability—were markedly lower in the infertile men with varicocele compared to the fertile group (*p* ≤ 0.001). Additionally, the proportion of sperm with abnormal morphology was significantly greater in men with varicocele than fertile men (Figure 2). Beyond conventional sperm assessments, this study also investigated sperm function concerning oxidative stress, chromatin packaging, and DNA damage. As demonstrated in Figure 2, levels of lipid peroxidation (measured via Bodipy Probe) and ROS (assessed using DCFH‐DA staining) were significantly higher in sperm from men with varicocele compared to the fertile group. Chromatin structure and DNA damage were further analyzed using three distinct tests: Aniline Blue staining for residual histones, CMA3 staining for protamine deficiency, and SCSA for sperm DNA fragmentation. The results, presented in Figure 3, indicate that all three parameters were significantly higher in men with varicocele than in fertile men, pointing to impaired chromatin packaging and DNA integrity.

**Figure 4:**
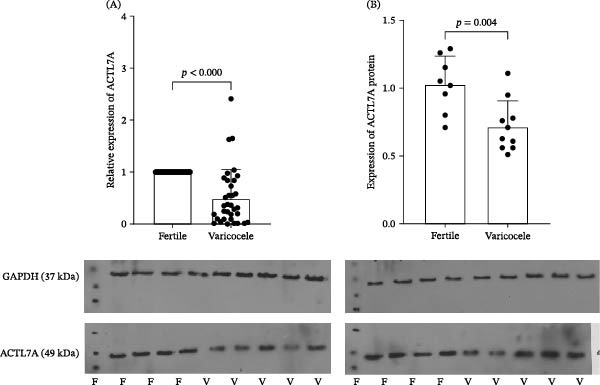
Comparison of sperm relative of ACTL7A mRNA (A) and protein (B) between infertile men with varicocele (V) and fertile controls (F). Data are presented as mean ± standard deviation (SD), with individual data points shown as scatter plots. Comparisons between groups were performed using an independent *t*‐test. *p*‐Values < 0.05 were considered statistically significant.

**Figure 5:**
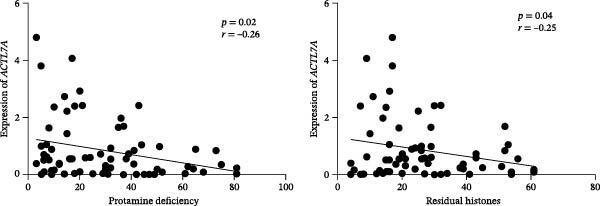
Pearson correlation analysis showed a weak but significant inverse association between ACTL7A expression at RNA with protamine deficiency (*r* = –0.26, *p* = 0.02) and residual histones (*r* = –0.25, *p* = 0.04). *p*‐Values < 0.05 were considered statistically significant.

The primary objective of this study was to assess the expression levels of the human *ACTL7A* gene and its corresponding ACTL7A protein in sperm. According to the findings shown in Figure 4, infertile men with varicocele exhibited significantly lower *ACTL7A* mRNA expression as well as reduced ACTL7A protein levels compared to fertile men. Furthermore, correlation analysis across the entire study population revealed a significant inverse association between *ACTL7A* transcript expression and both protamine deficiency (*r* = −0.26; *p* = 0.02) and residual histones (*r* = −0.25; *p* = 0.04). No significant correlations were observed between *ACTL7A* transcript levels and other sperm parameters or functional tests (Figure 5).

## 4. Discussion

This study provides novel insights into the role of ACTL7A in the pathophysiology of varicocele‐associated male infertility by demonstrating significantly reduced expression levels of the *ACTL7A* gene and its corresponding ACTL7A protein in sperm from infertile men with varicocele. These findings align with previous evidence indicating that ACTL7A, a testis‐specific actin‐related protein localized to the PT and acroplaxome complex, plays a central role in acrosome formation, sperm head shaping, cytoskeletal remodeling, and chromatin packaging during spermiogenesis [5, 7, 9]. Reduction of ACTL7A in varicocele likely reflects impaired sperm maturation due to elevated testicular temperature and oxidative stress. Consistently, sperm from affected individuals showed increased ROS and lipid peroxidation, indicating redox imbalance that may compromise chromatin integrity via disrupted protamine–histone exchange and elevated DNA fragmentation. Similarly, Karimi et al. [25] demonstrated that infertile men with high sperm DNA fragmentation exhibited significantly reduced ACTL7A expression, accompanied by increased ROS levels and impaired sperm motility, further supporting a relationship between ACTL7A deficiency, oxidative stress, and compromised sperm chromatin integrity.

Chromatin abnormalities—including increased residual histones, protamine deficiency, and elevated DNA fragmentation—were associated with decreased *ACTL7A* transcript expression, suggesting a potential involvement of ACTL7A in spermiogenic chromatin remodeling. Significant inverse correlations with markers of chromatin immaturity further support a direct or indirect role of ACTL7A in nuclear maturation. Although the observed correlations were modest, they were statistically significant and biologically meaningful, reflecting the contribution of ACTL7A to sperm chromatin remodeling within the multifactorial process of spermatogenesis.

Previous studies in animal models [26, 27] have shown that *ACTL7A* deficiency disrupts manchette formation, causes acrosomal detachment, abnormal sperm morphology, and impaired fertilization, partly via dysregulated autophagy through the PI3K/AKT/mTOR pathway. Although we did not directly assess these pathways, the reduced motility, viability, and abnormal head morphology observed in sperm from men with varicocele resemble the phenotypes of *ACTL7A*‐deficient models. Consistent with this, *ACTL7A* expression was significantly decreased in varicocele‐affected sperm, aligning with its reported role in autophagy modulation [26]. Supporting this notion, previous work demonstrated increased autophagic markers (*ATG7*, *LC3-II/LC3-I* ratio) in testicular tissue and sperm from varicocele models [28], with similar patterns of autophagy and apoptosis in human samples [29]. The concurrent reduction of ACTL7A and enhanced autophagic activity may reflect a compensatory response to oxidative and thermal stress, suggesting that ACTL7A is likely to be a direct or indirect contributor to sperm integrity via both cytoskeletal/nuclear remodeling and autophagy regulation, highlighting its multifaceted role in varicocele‐associated infertility.

Beyond its established role in spermatogenesis, ACTL7A has emerged as a potential biomarker for fertilization outcomes in ART and has been implicated in key fertilization processes, particularly oocyte activation [7, 30, 31]. Although ICSI is widely used to bypass natural barriers in male infertility, 1%–5% of cycles still experience total fertilization failure (TFF), often attributed to sperm‐derived oocyte activation deficiency (OAD) [32]. ACTL7A, alongside PLCζ, is essential for delivering oocyte‐activating factors and initiating calcium oscillations required for oocyte activation; mutations in *ACTL7A* (e.g., c.1118G >A: p.R373H; c.1204G >A: p.G402S) have been identified in infertile men and validated in animal models, demonstrating defects in cytoskeletal organization and mislocalization of activation‐related proteins [33–36]. The expression reduction of ACTL7A observed in varicocele individuals in our study could contribute to similar subclinical fertilization deficiencies, particularly in ART settings, by impairing acrosomal integrity and oocyte‐activating factor delivery. Varicocele, through oxidative stress, disrupted spermatogenesis, and impaired acrosomal architecture, can negatively influence the expression and localization of these SOAF—including ACTL7A. Therefore, the reduction of ACTL7A observed in varicocele patients in our study may predispose them to subclinical forms of OAD.

Moreover, ACTRT1 and SPACA1, which interact with ACTL7A within the pPT and acroplaxome, have also been implicated in structural acrosomal abnormalities and fertilization failure. In this regard, deletion of *ACTRT1* leads to reduction of *ACTL7A* and *Plcζ*, while *Spaca1* deficiency destabilizes acrosomal anchoring, mimicking the phenotypes seen in *ACTL7A*‐deficient sperm and highlighting the coordinated role of this protein network in sperm development [3, 26, 37]. As ACTL7A also emerges as a predictive biomarker of fertilization outcomes in ART, lower levels of this protein have been correlated with poor embryo development, increased embryonic arrest, and reduced fertilization success [7].

Cross‐species transcriptomic analyses, including in Bactrian camels, highlight a conserved role for *ACTL7A* in spermatogenesis, coregulated with key pathways such as TGF‐β, PI3K‐AKT, and WNT [38]. Integration of *ACTL7A* into complex regulatory networks—including long noncoding RNAs and microRNAs within a ceRNA framework—underscores its role in germ cell differentiation and transcriptional regulation. Additionally, ACTL7A also interacts with actin‐like proteins such as T‐ACTIN1, T‐ACTIN2, ACTRT2, CAPZA3, and MYO6, supporting its function in acrosome biogenesis, nuclear shaping, and sperm motility [38].

This study has several limitations. First, the sample size was relatively small, which may limit the generalizability of the results. Second, immunofluorescence analysis was not performed to confirm the subcellular localization of ACTL7A within spermatozoa. Finally, downstream signaling pathways of ACTL7A were not investigated, which could provide further mechanistic insights into its role in sperm development and function. Future studies addressing these limitations will strengthen our understanding of ACTL7A in male infertility.

## 5. Conclusion

Our findings show that ACTL7A expression is significantly reduced in sperm from men with varicocele, likely contributing to defective chromatin remodeling and abnormal sperm head formation. This is the first study linking varicocele‐associated oxidative stress to ACTL7A, integrating molecular, structural, and functional aspects of male infertility. ACTL7A may serve as a biomarker of testicular dysfunction, and future studies should investigate whether interventions such as antioxidant therapy, varicocelectomy, or varicocele grade and duration affect sperm quality and reproductive outcomes.

## Author Contributions

Mohammad H. Nasr‐Esfahani and Marziyeh Tavalaee made substantial contributions to the study, including conceptualization, study design, coordination, data analysis, manuscript drafting, revision, and final approval. Sahar Tahamtan contributed to the material preparation, data collection, and experimental procedures. Mohsen Forouzanfar was the supervisor of student. Mojtaba Jafarinia was the faculty advisor

## Funding

This work was financially supported by the Iran National Science Foundation (INSF, project number 4015398) and the Royan Institute for Reproductive Biomedicine Foundation (project number 402000004).

## Disclosure

The funding bodies had no role in study design, data collection, analysis, or manuscript preparation. All scientific content and original writing remain entirely the authors’ own.

## Ethics Statement

This study was approved by the Royan Institute’s Ethics Review Board under the ethical code IR.ACECR.ROYAN.REC.1402.021.

## Conflicts of Interest

The authors declare no conflicts of interest.

## Data Availability

The data in the current study are available from the corresponding author upon reasonable request.
